# A Rare Case of Spontaneous Pneumomediastinum in a Female After Shouting: A Literature Review

**DOI:** 10.7759/cureus.30894

**Published:** 2022-10-31

**Authors:** Mohammad Al-Hurani

**Affiliations:** 1 Department of General Surgery, Urology and Anesthesia, Faculty of Medicine, The Hashemite University, Zarqa, JOR

**Keywords:** emergency medicine and trauma, chest pain in the young, pulmonary disease, adult thoracic surgery, spontaneous pneumomediastinum (spm)

## Abstract

Spontaneous pneumomediastinum (SPM) is a rare self-limiting benign condition that manifests as free air in the mediastinum without any underlying etiology. This under-reported diagnosis occurs usually in young males, with few reports in females. Here, we present a case of a 17-year-old healthy female who presented to the emergency department (ER) with shortness of breath (SOB) and chest pain preceded by an episode of shouting. And she was identified to have SPM and subcutaneous emphysema (SE).

## Introduction

Spontaneous pneumomediastinum (SPM) is a self-limiting disease with benign course that usually resolves spontaneously without any active surgical or medical intervention. This entity is more common in males as compared to females, with hospital admissions ranging from one case per 800 admissions to one case per 42,000 admissions [1].

Usually, it results from alveolar rupture, which happens due to a change in the pressure gradient between the alveoli and the lung interstitium. Later, air tracks through the bronchovascular sheaths until it reaches the mediastinum [1]. The rupture can be induced by any factor that elevates the intrathoracic pressure (e.g., shouting and labor). Here, we report a case of a teen female with negative past medical and surgical history that presented with acute onset of shortness of breath (SOB) and chest pain associated with neck swelling after an episode of shouting.

## Case presentation

We present a case of a 17-year-old healthy female who presented to the ER complaining of a sudden sharp chest pain of one-day duration after an episode of intense shouting. The pain was associated with SOB and neck swelling. However, she denied any history of cough, vomiting, hoarseness of voice, difficulty in speaking or swallowing, or constipation. Furthermore, she didn’t have any history of trauma or any intense exercises. Also, she did not undergo any recent dental procedures. Furthermore, she denied tobacco or drug usage. Additionally, she has no previous history of childhood asthma.

On examination, she was alert and hemodynamically stable; her blood pressure was 130/70 mmHg, heart rate was 84 beats/minute, respiratory rate was 17 breaths per minute, oxygen saturation on room air was 93%, and she was afebrile. The rest of her physical examination was unremarkable except for minimal subcutaneous emphysema at the root of the neck and supraclavicular fossa. Additionally, she has no marfanoid features.

**Figure 1:**
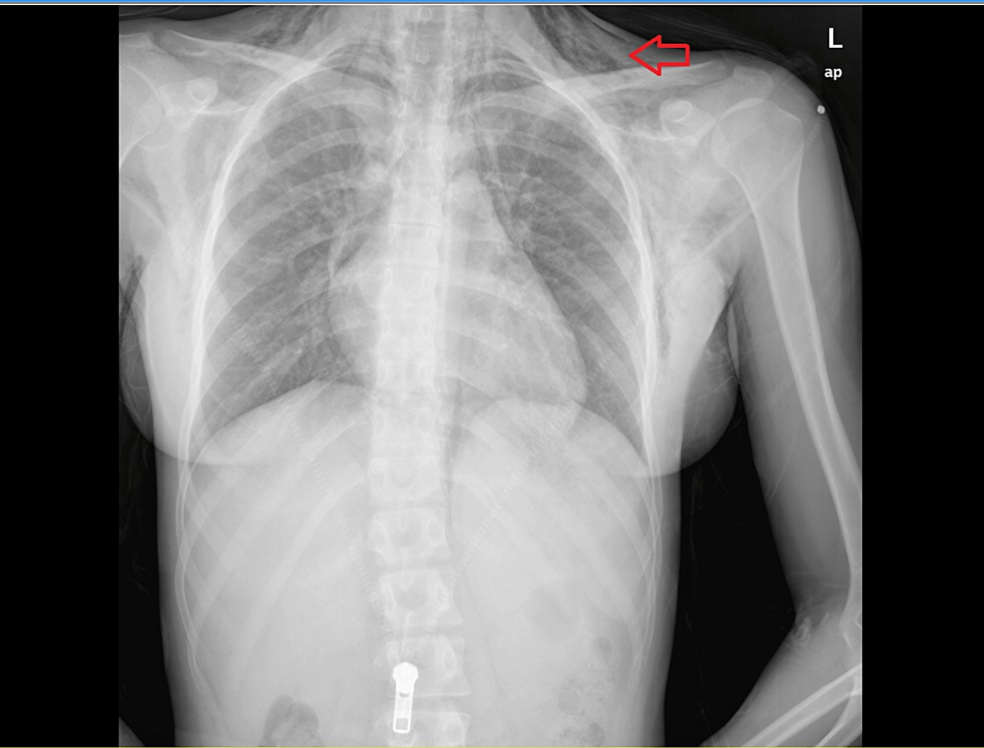
Chest radiography (CXR) with anterior-posterior projection. The image shows air in subcutaneous tissue relevant to subcutaneous emphysema (SE) (arrow).

After initial assessment, relevant laboratories - including complete blood count, c-reactive protein, and kidney function test - were ordered, and all were within normal limits. Initially, chest radiography (CXR) was ordered; CXR showed free air tracking in the subcutaneous tissue relevant with SE but without pneumothorax (Figure 1).

**Figure 2:**
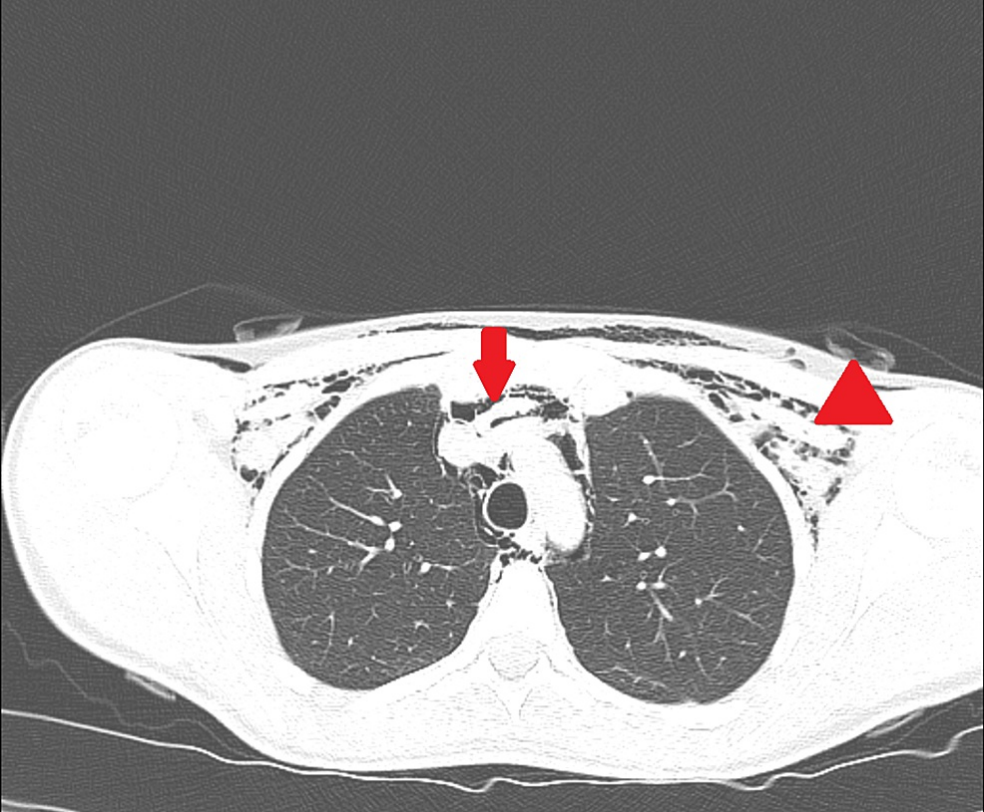
Cross-sectional CT scan of chest. The image shows pneumomediastinum (arrow) as free air anterior to the arch of aorta and trachea. Furthermore, it shows subcutaneous emphysema (SE) (arrowhead) beneath the pectoralis major muscle and skin.

Subsequently, computed tomography scan (CT scan) of neck and chest was ordered and revealed pneumomediastinum without pneumothorax but associated with SE in the neck and chest (Figure 2).

The patient was given supplemental oxygen and analgesia in the ER but without any empirical antibiotic, and she was observed there for a couple of hours, during which she remained stable. Then she was discharged from the ER with advice and scheduled to visit the outpatient clinic two days later. In the outpatient clinic, she declared improvement in her symptoms, in addition to decrease air in subcutaneous tissue of neck and upper chest on clinical examination. One week later, she visited the clinic again, and her symptoms had completely disappeared.

## Discussion

SPM is a clinical description of abnormal existence of air in the mediastinum without being associated with any underlying pathology [1-4]. SPM may result from air escaping from ruptured alveoli and is usually associated with SE. In addition, it may be associated with crepitus heard on auscultation of the heart (Hamman’s sign). This phenomenon was first described by Dr. Louis Hamman. And he reported the first case in 1939; later, he reported seven cases over a five-year period [5,6].

The incidence of SPM increased, and this increment in number of documented cases could be attributed to the significant advancement in radiological imaging technology. Despite that, it is still considered a rare clinical condition [1,7]. SPM is more common in males as compared with females, and most patients are tall, healthy, thin males with ages ranging from 20 to 25 years [6].

SPM follows a sudden rise of the intrathoracic pressure due to any trigger, which in turn, leads to alveolar rupture [6]. Consequently, air escapes toward the hilum and mediastinum. Accumulation of air in the mediastinum will elevate the pressure in this compartment. Subsequently, air dissects into cervical fascia leading to the development of SE; thus, preventing the development of tension pneumomediastinum which is considered fatal [6]. Sadarangani et al. mentioned that the changes in pressure could be attributed to the following three factors [8]: increased alveolar pressure (Valsalva maneuver), decreased pleural pressure (Muller’s maneuver), or decreased interstitial pressure (intense breathing) [2,3,5].

Most patients present with retrosternal pleuritic chest pain with SOB, these symptoms could associate with other symptoms like dysphagia, neck pain and swelling, and hoarseness of voice [5]. On the presentation, patients are usually hemodynamically stable, and the physical examination is usually normal except for some SE which may be felt in the neck and upper chest. Additionally, small group of cases could have positive Hamman’s sign which is considered pathognomonic [8]. When the clinician predicts this entity, CXR with posterior-anterior projection (PA) is not enough since 30-50% of cases can be failed to spot [6]. Although lateral CXR is essential for the diagnosis to be recognized, a retrosternal free air may be the only finding in some cases, still some cases were missed [3]. Hence, to confirm the diagnosis and to figure out the extent and severity of the disease, CT scan remains the gold standard in radiological imaging.

Usually, the management of this condition is conservative. Hospitalization, 100% oxygen supplement, and prophylactic antibiotics are debatable [8]. Some clinicians prefer to observe the patient closely and give 100% oxygen and prophylactic antibiotics. However, no compelling evidence supports this practice [8]. In this report, the patient was observed in the ER for a couple of hours and sent home with advice to seek medical help in case of any deterioration in her symptoms. Furthermore, needle decompression should be avoided since it risks contaminating the subcutaneous tissue and the mediastinum [6].

We've conducted a review of the literature using Medical Subjects Headings (MeSH) terms "pneumomediastinum" and "mediastinal emphysema" in the PubMed database. Then we excluded all cases related to coronavirus disease 2019 (COVID-19). Furthermore, we only included cases of SPM that happened after shouting (Table 1). In our review of the literature, we found 12 cases of SPM happened after shouting; seven out of 12 were documented in one center in Japan after analyzing 71 cases of SPM over 15 years [2]. Unfortunately, Yamairi et al. didn’t figure out the exact number of female patients out of the seven cases that have been documented [2]. Most cases were males, and only one case was documented as a female. Additionally, most cases happened in young patients. Furthermore, most of that patients were admitted and treated conservatively. Interestingly, in our case report, the patient was not admitted to the hospital.

**Table 1:** Literature review: shouting-induced pneumomediastinum.

Author	Year	Country	Sample size	Age (years)	Sex	Treatment
Yamairi et al. [2]	2020	Japan	Seven	19.3±6.4	75% males	Conservative
Slaughter and Roppolo [4]	2016	USA	One	18	Female	Conservative
Singla et al. [5]	2012	USA	One	25	Male	Conservative
Jones and Kundrotas [6]	2011	USA	One	20	Male	Conservative
Paixão et al. [7]	2021	Brazil	One	50	Male	Conservative
Sherrier and Lizardo [9]	2019	USA	One	19	Male	Conservative

Additionally, we excluded one case reported by Kim et al., where the report documented a case of pneumomediastinum that happened after pharyngeal perforation caused by shouting; hence, we can not consider it as spontaneous [3].

## Conclusions

SPM is a self-limited benign condition with uneventful recovery in most cases. Careful history-taking and physical examination for this group of patients can save us from unnecessary investigations and disproportionate management. The decision to admit the patient is still questionable, further studies are needed to investigate the safety of discharge from emergency department. However, many factors will affect our decision; one of them is the distance between the healthcare provider and the place of patient's residency. In our case, the patient was living near the hospital, and she was given strict advice to visit ER in case of any deterioration.
